# Enhancing PEM fuel cell efficiency with flying squirrel search optimization and Cuckoo Search MPPT techniques in dynamically operating environments

**DOI:** 10.1038/s41598-024-64915-7

**Published:** 2024-06-17

**Authors:** Assala Bouguerra, Abd Essalam Badoud, Saad Mekhilef, Badreddine Kanouni, Mohit Bajaj, Ievgen Zaitsev

**Affiliations:** 1 Automatic Laboratory of Setif, Electrical Engineering Department, University Ferhat Abbas of Setif 1, City of Maabouda, Algeria; 2 School of Software and Electrical Engineering, Swinburne University of Technology, Melbourne, Australia; 3 Power Electronics and Renewable Energy Research Laboratory (PEARL), Department of Electrical Engineering, University of Malaya, 50603 Kuala Lumpur, Malaysia; 4 Department of Electrical Engineering, Graphic Era (Deemed to Be University), Dehradun, 248002 India; 5 Hourani Center for Applied Scientific Research, Al-Ahliyya Amman University, Amman, Jordan; 6 Graphic Era Hill University, Dehradun, 248002 India; 7 Department of Theoretical Electrical Engineering and Diagnostics of Electrical Equipment, Institute of Electrodynamics, National Academy of Sciences of Ukraine, Peremogy, 56, Kyiv-57, 03680 Ukraine; 8 Center for Information-Analytical and Technical Support of Nuclear Power Facilities Monitoring of the National Academy of Sciences of Ukraine, Akademika Palladina Avenue, 34-A, Kyiv, Ukraine

**Keywords:** Boost converter integration, Cuckoo Search (CS), Dynamically operating environments, Flying Squirrel Search Optimization (FSSO), Maximum power point tracking (MPPT), PEM fuel cell, Energy science and technology, Engineering, Mathematics and computing

## Abstract

This study looks into how to make proton exchange membrane (PEM) fuel cells work more efficiently in environments that change over time using new Maximum Power Point Tracking (MPPT) methods. We evaluate the efficacy of Flying Squirrel Search Optimization (FSSO) and Cuckoo Search (CS) algorithms in adapting to varying conditions, including fluctuations in pressure and temperature. Through meticulous simulations and analyses, the study explores the collaborative integration of these techniques with boost converters to enhance reliability and productivity. It was found that FSSO consistently works better than CS, achieving an average increase of 12.5% in power extraction from PEM fuel cells in a variety of operational situations. Additionally, FSSO exhibits superior adaptability and convergence speed, achieving the maximum power point (MPP) 25% faster than CS. These findings underscore the substantial potential of FSSO as a robust and efficient MPPT method for optimizing PEM fuel cell systems. The study contributes quantitative insights into advancing green energy solutions and suggests avenues for future exploration of hybrid optimization methods.

## Introduction

Energy permeates all aspects of our world, playing a fundamental role in the construction and functioning of various entities. In particular, electricity serves as a cornerstone for residential, commercial, and individual activities, with its significance only expected to grow with technological advancements^1^. In response to the escalating demand for sustainable energy solutions, environmentally conscious infrastructures are embracing diverse storage mechanisms to harness renewable energy sources. Especially in the realm of fuel cells (FCs) and their diverse applications, the utilization of sustainable hydrogen from natural reservoirs is gaining traction^2^. In this situation, proton exchange membrane fuel cells (PEMFCs) have become a popular technology because they are small, can start up quickly, work at low temperatures, and consistently produce electricity. Widely deployed in electric vehicles, energy-efficient systems, and beyond, PEMFCs are pivotal in advancing green energy initiatives^3^. Things like the temperature of the cell, the amount of water in the membrane, and changes in the relative pressures of oxygen and hydrogen gases complicate the electrical performance of PEMFCs. Similar to solar panels, fuel cells exhibit a non-linear relationship between current and voltage, wherein power generation peaks at a specific operating point known as the Maximum Power Point (MPP)^4^. In order to consistently get the most power out of fuel cells, you need a way to track the MPP even when the operating conditions change. This is where the Maximum Power Point Tracking (MPPT) strategy comes in^5,6^. In the past few years, a lot of research has gone into improving MPPT techniques^7^. These techniques aim to make systems more efficient by speeding up the reach of the MPP, improving performance around this point, and making controllers more accurate so they can respond well to changes in the outside world^8,9^. A comprehensive review of relevant literature underscores the significance of MPPT strategies in various applications^10,11^. Specifically, the Perturb and Observe (P&O) method has become a popular way to study hydrogen-powered vehicles. It is faster and more accurate than incremental conductance (IC) techniques^12^. Moreover, conventional hill climbing (HC) methods, alongside P&O algorithms, find widespread utilization in optimizing PEMFC operation under diverse environmental conditions.

Meta-heuristic optimization methods^13^ that use biological and natural processes have shown to be very good at solving hard optimization problems in a wide range of areas. Furthermore, these methods possess the capability to conduct global searches and exhibit robustness against noise while also demonstrating adaptability to changing circumstances. Meta-heuristic algorithms, which use swarm intelligence, evolutionary computation, and collective behavior, look like a good way to improve the MPPT performance of PEMFCs. We can improve the efficiency of energy extraction, ensure system stability, and effectively mitigate environmental variation by implementing sophisticated optimization techniques. In order to address the inherent difficulties associated with conventional approaches, we are currently investigating meta-heuristic optimization techniques for MPPT in fuel cells^14,15^. Prior research has extensively employed meta-heuristic concepts to pinpoint optimal operating points. Reference^16^ advocates for sustaining the ideal output of FCs through high-order mode control with sliding techniques. Reference^17^ proposes an approach that uses a PID-controlled device, optimized through gray wolf optimization, to adjust the duty cycle of the boost converter in response to environmental changes. Combining fuzzy logic and particle swarm optimization, Luta et al.^18^ introduce an innovative MPPT approach. Furthermore,^19^ introduces a Modified Particle Swarm Optimization (MPSO) technique for MPPT optimization. Fathy et al.^20^ use the salp-swarming approach (SSA), a meta-heuristic optimization method, to get the most power out of the proton exchange membrane. Harrabi et al.^21^ suggest a way to control MPPT in renewable energy installations that combines incremental conductivity (IC) and fuzzy logic (FL) methods to adapt to changing weather conditions, especially when using a buck-converter control scheme. Additionally,^22^ suggests the incorporation of fuzzy logic controllers to address rapid temperature fluctuations during MPPT. Harrag and Bahri^23^ use simulation-based approaches to optimize MPPT using an artificial neural network (ANN) with incremental conductive-sized steps. Lastly, Derbeli et al.^24^ present an MPPT technique utilizing the backstepping approach, offering another avenue for enhancing fuel cell efficiency and performance. In^25^ the aouthers proposed SOANN MPPT,the aouthors proposed a novel energy valley optimizer (EVO)^26^, in^27^ proposed initialization approach is applied to the simplest conventional MPPT technique, which is the perturbation and observation (P&O) algorithm, to show the enhancement in performance without the need to introduce any complex processes in^28^ evolutionary NN-based MPPT control technique.

We can combine FSSO and CSO with other optimization methods or traditional Maximum Power Point Tracking approaches to enhance the optimization results in fuel cell systems. This approach combines meta-heuristic algorithms with conventional techniques, improving robustness, convergence speed, and adaptability to different operating conditions. We can use ensemble learning methods such as stacking, boosting, and bagging to construct diverse optimization ensembles, thereby enhancing overall performance^29^. A comprehensive evaluation of hybrid and ensemble methods is essential to determining the most efficient strategies and guiding future research in this area^30^. Also Ant Colony Optimization and Fuzzy Logic^31^, An innovative grey wolf optimizer with Nelder–Mead search method^32^, Integration of a Hybrid Incremental Conductance Integral Backstepping Algorithm^33^, Conjugate Gradient (CG) to address the problem of hard reasonable considering both dynamic and steady-state performance in some existing MPPT algorithms^34^, Optimized MPPT model for different environmental conditions to improve efficacy^35^, Dung Beetle Optimization Algorithm (DBO)^36^, investigates the tracking mechanism in HOM^37^, Improved coot optimizer algorithm^38^, A novel population based maximum point tracking algorithm to overcome partial shading issues in solar photovoltaic technology^39^. Metaheuristic based comparative MPPT^40^, proposes an adaptive integral sliding mode MPPT^41^, the authors present a new Maximum Power Point Tracking (MPPT) controller. This controller is based on a modified version of the heterogeneous multi-swarm Particle Swarm Optimization (PSO) algorithm. They also include an adaptive factor selection technique called FMSPSO. The purpose of this controller is to address the limitations of prior approaches and compare its performance with that of traditional PSO^42^.

PEM hydrogen fuel cells represent a revolutionary advancement in environmentally sustainable power generation, renowned for their exceptional efficiency and adaptability^43,44^. Optimizing MPPT in Proton Exchange Membrane (PEM) fuel cells presents challenges due to the dynamic nature of operating conditions. Maximum Power Point Tracking efficiency in PEM fuel cells is difficult to achieve because the electrochemical reactions in these cells are complex and non-linear. There are many unknowns because of all the environmental variables, which change over time, including temperature, pressure, and the water's membrane content^45^. Traditional MPPT methods often fall short of adapting to changing environmental factors, leading to suboptimal energy conversion efficiency. This gap underscores the need to explore innovative optimization techniques, such as Flying Squirrel Search Optimization (FSSO) and Cuckoo Search (CS), to enhance MPPT performance in PEM fuel cells. Additionally, the limited exploration of advanced algorithms and the requirement for heightened adaptability in dynamic operating environments further emphasize the need for comprehensive investigation. So, the goal of our study is to find out how well FSSO and CS algorithms work and how they might be able to improve MPPT in PEM fuel cells under different conditions. This will help make energy conversion systems that are more efficient and flexible.

This research aims to investigate whether the MPPT and Flying Squirrel Search Optimization algorithms can enhance proton exchange membrane FCs. The primary objectives of our research were to determine how well FSSO and CS strategies optimized MPPT under varying settings and circumstances, and to measure how well they scaled. We are aiming to find out the answers to these research questions: Can you tell me how well the FSSO and CS algorithms optimize Maximum Power Point Tracking when the temperature and pressure are changing? We think that the FSSO and CS algorithms will show that traditional MPPT methods have slower convergence and less effective tracking in a variety of environmental conditions. Regarding the second question, it is important to know whether the FSSO and CS algorithms can scale well for MPPT across a range of PEM fuel cell system sizes and configurations. Based on our hypothesis, the FSSO and CS techniques should be able to scale well, meaning that they keep convergence speed and optimization quality constant no matter how big or small the system gets. Why are FSSO and CS algorithms better at MPPT than other meta-heuristic optimization methods? MPPT in PEM fuel cells is an important optimization problem, and we predict that the FSSO and CS algorithms will outperform competing meta-heuristic optimization approaches in these respects. This research aims to enhance sustainable energy generation by examining the practicality and efficiency of the FSSO and CS techniques for Maximum Power Point Tracking in PEMFCs. We hope to address the raised questions and seek clarifications.

Both PEM fuel cells and MPPT optimization have greatly benefited from this study's substantial contributions to the literature. To begin, we investigate the viability and efficacy of advanced algorithms like FSSO and CS in optimizing MPPT under different operating conditions by analyzing their efficacy. Furthermore, our research enhances our comprehension of MPPT techniques' adaptability to diverse environments, thereby facilitating their practical application. By comparing how well FSSO and CS work with standard MPPT methods, we also provide useful analysis and comparisons that help people choose the best optimization methods for PEM fuel cell systems. Contributing significantly to the ongoing development of highly effective and environmentally friendly energy conversion technologies, this study lays the framework for future research on hybrid optimization methods and practical implementation approaches. In addition to paving the way for further advancements in the development of clean energy solutions, our results contribute to the current body of knowledge regarding the optimization of PEM fuel cells.

## System description

**Figure 1:**
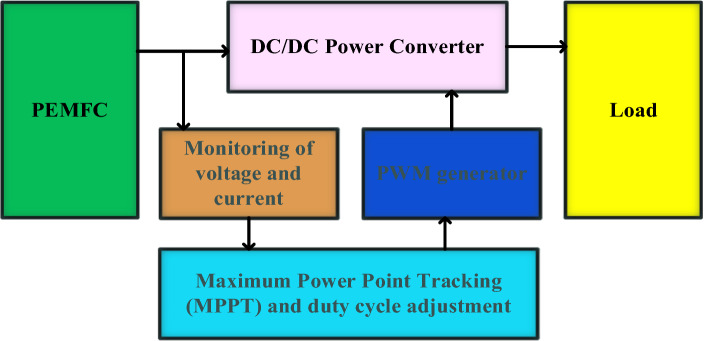
Schematic of a PEMFC system based on MPPT.

The PEMFC (Proton Exchange Membrane Fuel Cell) system utilizes a DC-to-DC power converter to control its current and voltage generation. Surveillance systems consistently supervise these variables, delivering up-to-date information for calculating the Maximum Power Point Tracking. With the help of a pulse width modulation (PWM) generator, the method adjusts the duty cycle of the converter to boost the fuel cell's efficiency by operating at its maximum power point. The power delivery system distributes power to the load, ensuring optimal efficiency while maximizing power reuse. Figure 1 displays a schematic of a PEMFC system based on MPPT.

## System dynamics in fuel cell technology

### Understanding fuel cell systems

#### Components and operation

The most effective way of producing electricity is that fuel cells transform the combustible potential of hydrogen into power and heat^46^. Conventional fuel cells consist of an anode, cathode, and electrolyte surrounded by two electrodes. The electrolyte facilitates protons' movement. Electrochemical cells, also known as galvanic cells, generate electricity through the spontaneous oxidation and reduction of atomic components. The main components are membrane electrode assemblies (MEAs) and gas diffusion layers. An electrode known as the anode facilitates the movement of hydrogen, while a catalyst accelerates the movement of oxygen via a cathode. Platinum's superior electro-catalytic properties make it a popular hydrogen fuel ion separation choice. The electrolytic membrane prevents electrons from flowing freely, so they must pass into electricity using an extra connection. In FC, the responses occur in the following way^47^:

1 $$ {\text{Electrochemical anode}}{:}\;H_{2} = 2H^{ + } + 2e^{ - } $$

2 $$ {\text{Electrochemical cathode}}{:}\;\frac{1}{2}O_{2} (g) + 2H^{ + } + 2e^{ - } = H_{2} O $$

3 $$ {\text{Overall reaction}}{:}\;2H_{2} + O_{2} = 2H_{2} O + Electricity + Heat $$

**Figure 2:**
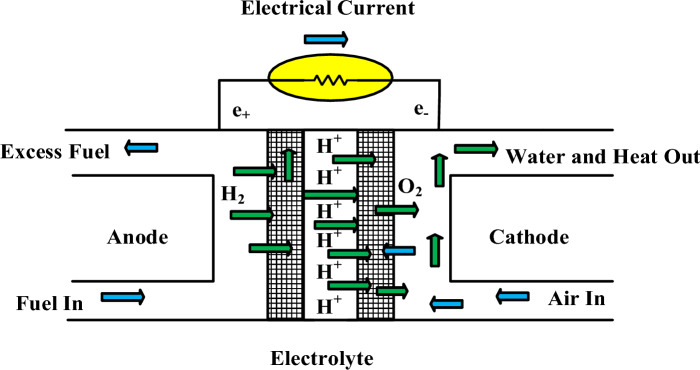
A schematic of a PEM fuel cell.

Like batteries, until a hydrogen fuel is available, as shown by these reactions. Figure 2 displays a schematic of a PEM fuel cell.

#### PEMFC modelling

**Figure 3:**
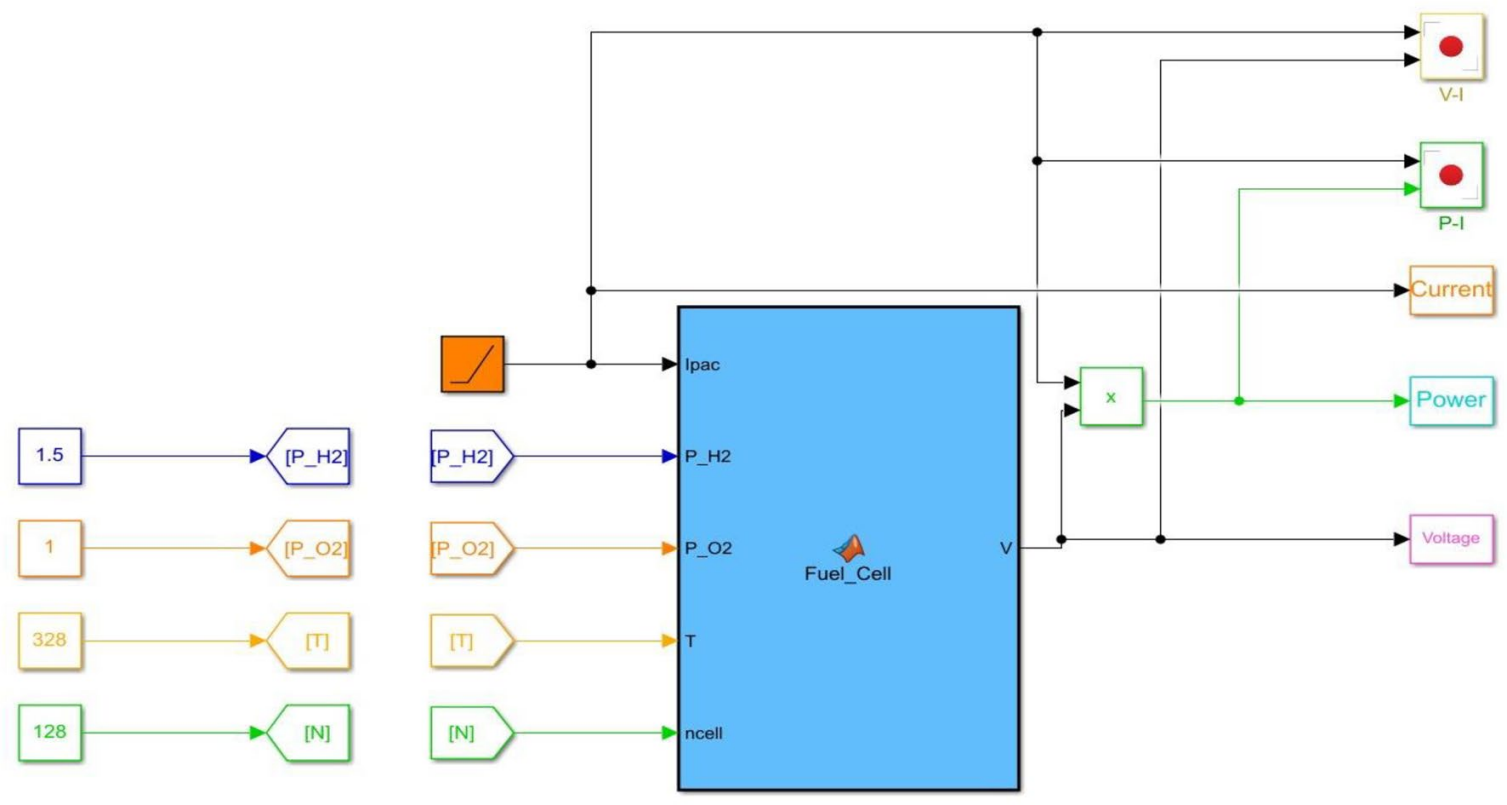
Simulink model of fuel cell power and voltage current.

**Figure 4:**
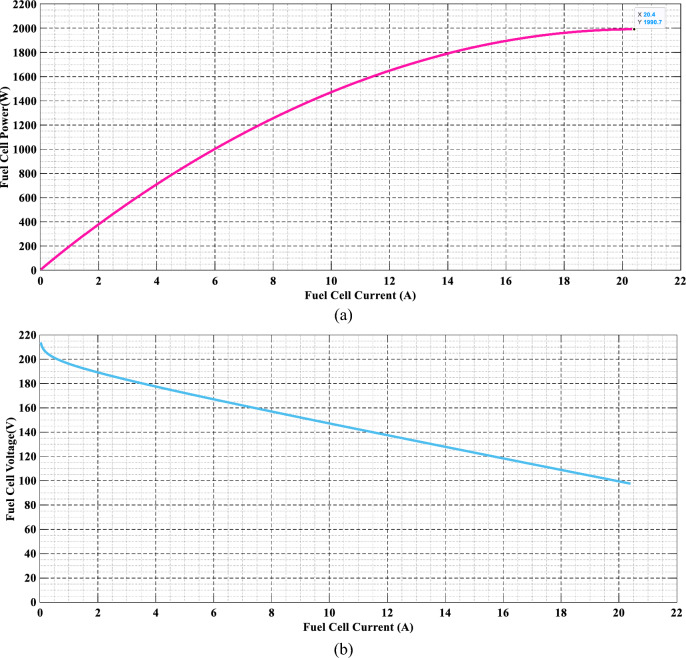
(**a**) The fuel cell's power-current and (**b**) its voltage-current trait.

In a steady state, a polarization curve can show an association between a hydrogen cell's current supply and voltage density^48^. Variables influencing this curve include cell heat, hydrogen and oxygen partial pressures, and membrane water content^49^ fuel cell parameters, including temperature $\left( T \right)$. Hydrogen partial $\left( {P_{{H_{2} }} } \right)$ and particulate oxygen pressure $\left( {P_{{O_{2} }} } \right)$ affect both voltage-current and power-current curves, respectively^49^. Figure 3 illustrates the process of creating a plot for a fuel cell's power-current and voltage-current curves, while Fig. 4 presents a demonstration of fuel cell power and voltage-current graphs.

FC has drooping characteristics due to voltage drops produced by activation, concentration, and ohmic losses^50^. Those dips, as explained by Larminie, Dicks, and McDonald in 2003^51^, are what define the FC output voltage.

4 $$ {\text{Vcell = ENernst - Vact - Vohmic - Vcon}} $$

The energy cell membrane is $V_{cell}$ $V_{act}$ the activation energy (caused by the chemical reaction), and V_con_ is the concentration loss from water overflow in the fuel cell catalyst. $V_{ohmic}$ is the Ohmic loss resulting from hydrogen cell-intrinsic resistance. The voltage at which only one cell changes to an open circuitry output direction is referred to as $V_{Nernst}$, also known as its thermal-electric capacity, as established by Larminie, Dicks, and McDonald^51^ and Wang et al.^52^.

5 $$ {\text{ENerst = }}\frac{\Delta G}{{2F}} + \frac{\Delta S}{{2F}}(T - T_{0} ) + \frac{RT}{{2F}}[\ln (PH_{2} ) + \frac{1}{2}\ln (PO_{2} )] $$

where: $\Delta S$: entropy change, $\Delta G$: Gibbs liberated energy $\left( {\text{J/mol}} \right)$, $R$: The global gas factor, $F$: Faraday unchanged, $T$: The cell's temperature during operation.

6 $$ {\text{ENerst = 1}}{.229 - 8}{\text{.5}} \times {10}^{ - 4} (T - 298.15) + 4.308 \times {10}^{ - 5} (\ln (Ph_{2} ) + 0.5\ln (PO_{2} ) $$

According to Egiziano et al.^28^, the activation voltage drop $V_{act}$ is provided as follows:

7 $$ Vact = \left[ {\xi_{1} + \xi_{2} + \xi_{3} T\ln (CO_{2} ) + \xi_{4} T\ln (IFC)} \right] $$

The characteristic coefficients of the Fuel Cell are denoted as $\xi_{i} (i = 1 - 4)$, and the current through $FC$ is denoted as $I_{FC}$, $CO2 \, \left( {atm} \right)$ representing oxygen concentration ${\text{mol/cm}}^{ - 3}$, as determined by the equation below:

8 $$ CO_{2} = \frac{{PO_{2} }}{{(5.08 \times 10^{6} ) \times \exp ( - 498/T)}} $$

The following is how to calculate $V_{ohmic}$ an Ohmic overvoltage drop.

9 $$ Vohmic = I_{FC} (R_{M} + R_{C} ) $$

The operating temperature unaffected the lead contact resistance, which is denoted as $R_{C}$ constant $FC$. Equation (10) represent $R_{M}$ s the PEMFC's resistance.

10 $$ R_{M} = \frac{{r_{m} t_{m} }}{A} $$

11 $$ r_{m} = \frac{{181.6\left[ {1 + 0.03(I_{FC} /A) + 0.0062(T/303)(I_{FC} /A)^{2.5} )} \right]}}{{\left[ {\lambda_{m} - 0.634 - 3(I_{FC} /A)} \right]\exp [(4.18(T - 303/T))]}} $$

In this equation:

$$ r_{m}$$: represents the electrolyte membrane's resistance (in ohms per centimeter), $$t_{m}$$: denotes the dimension of the membrane (cm), $$A$$: represents the active surface space of the fuel cell (70 cm^2^), $$\lambda_{m}$$: represents the membrane's water content.

The voltage loss $V_{con}$ caused by excessive water flooding in the $FC$ catalyst may be stated as:

12 $$ V_{con} = - B\ln \left( {1 - \frac{j}{{j_{\max } }}} \right) $$

With: $J_{\max }$: represents the Maximum current density, $B$: denotes an Empirical constant that depends on the type of battery and its operation, *j*: denotes the maximum current, The fuel cell acquires its combined energy and voltage supply in the following manner:

13 $$ V_{FC} = N_{FC} V_{cell} $$

14 $$ P_{FC} = V_{FC} I_{FC} $$

**Figure 5:**
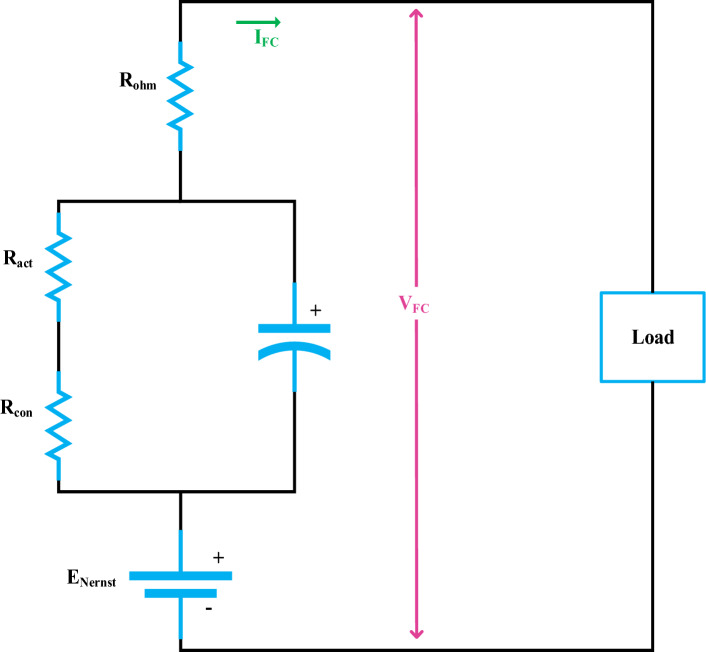
Diagram illustrating PEM fuel cell circuitry.

The final voltage, power, and current of a PEMFC are denoted as $$V_{FC}$$$$P_{FC}$$ and $$I_{FC}$$_,_ while the global fuel cell number is represented as $$ N_{FC}$$. The flowing Fig. 5 demonstrates a circuit diagram model of a PEM fuel cell.

### Understanding boost converter functionality

**Figure 6:**
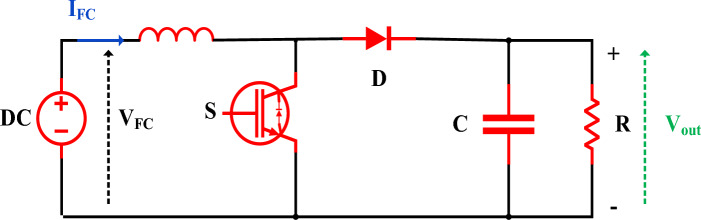
Boost converter circuit.

It is usual practice to use a tuning stage between the PEMFC and the load to attain the highest power output at all times^53^. A boosting converter enhances system efficiency by adjusting the duty cycle to regulate the hydrogen cell's highest power. The figure depicts a complete DC-to-DC boost converter structure with capacitance $C$, inductance $L$, diode $D$, resistance to load $R$, and switching semiconductor $S$. Figure 6 displays the boost converter circuit. Insufficient capacitance and inductance result in excellent current disturbance and voltage waves, making it essential to select the appropriate values to maintain a uniform output. The equation for the boost converter can be found in references^54–58^.

15 $$ \left\{ \begin{gathered} \frac{{dI_{L} }}{dt} = \frac{{V_{out} }}{L}(D - 1) + \frac{{V_{FC} }}{L} \hfill \\ \frac{{dV_{out} }}{dt} = \frac{{I_{L} }}{C}(1 - D) + \frac{{V_{out} }}{RC} \hfill \\ \end{gathered} \right. $$

where: $V_{FC}$ : Signifies the resultant voltage, $V_{out}$ : Stands for target voltage, $I_{L}$: represents the current of PEMFC, $D$ : represents the duty ratio.

The duty cycle can be calculated using the following formula:

16 $$ D = \frac{{V_{FC} }}{1 - D} $$

### Understanding the optimization techniques used

#### Cuckoo Search Optimization (CSO)

Cuckoos, including Ani and Guira, are renowned for their aggressive mating behavior and beautiful songs. To increase their egg-hatching chances, they may remove eggs from other birds' nests. Cuckoo birds, like the tapera, engage in brood parasitism by imitating their hosts' plumage. Females search for host species with similar nesting and egg production characteristics before selecting an ideal nest. If tricked, eggs are removed, or the nest is destroyed, forcing the birds to find a new nest^59,60^.

**Figure 7:**
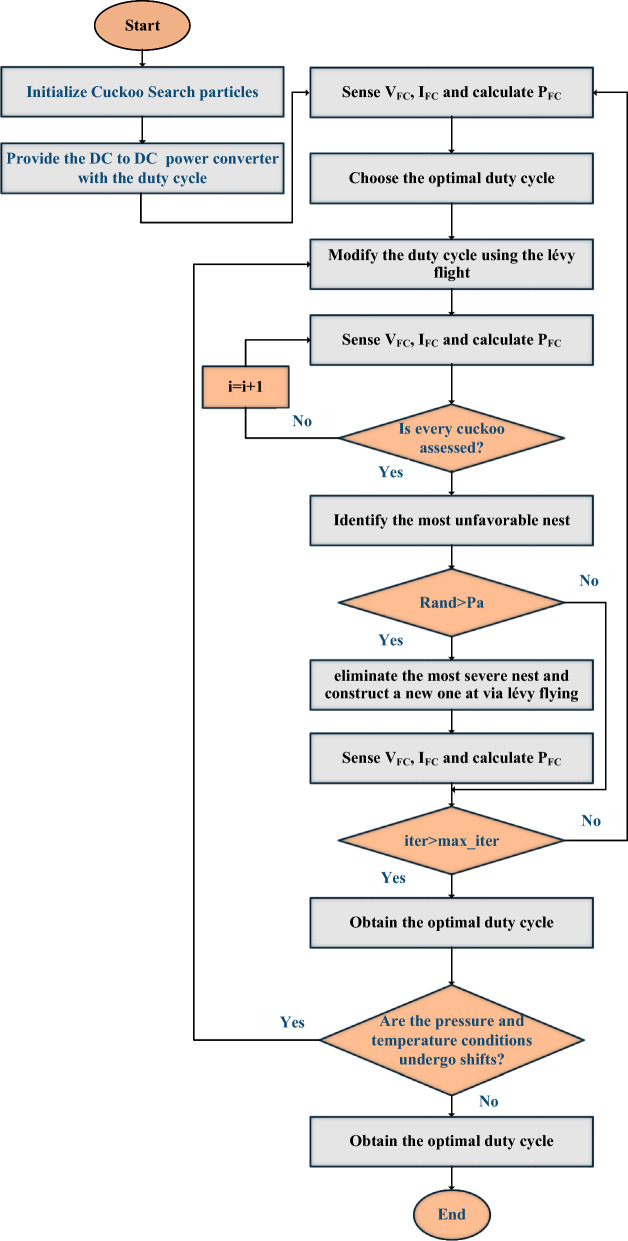
CSO MPPT algorithm flowchart.

The CS-MPPT Method is a cuckoo algorithm that randomly selects a nest to lay a single egg. We place the best eggs in surrogate nests reserved for the next generation. Only cuckoos have the ability to lay the egg, and they determine the likelihood of the host bird finding it. If the host bird finds the eggs, it can either eat them or destroy the nests. The Pa fraction is the simplest approximation for the final assumption. Next, nests replace the current solutions with fresh, randomly selected solutions. Future calculations and outcomes incorporate the chosen substandard nests, each egg representing a potential strategy. The primary step in cuckoo reproduction is finding the ideal nest, which is the same process as foraging and searching. Reynolds and Frey developed the Lévy flight model, which illustrates how fruit flies navigate their environment using straight lines and sudden 900-degree turns. We apply this behavior when optimizing for various problems, as the length of a Levy flight's steps follows a probability distribution^61–63^. Figure 7 displays the flowchart of the CSO MPPT algorithm.

#### A flying squirrel search optimization (FSSO) approach

##### Analyzing the FSS optimization technique

The flying squirrel search approach emulates the active seeking behavior of southern FSs and the floating movements of southern squirrels who fly. The result vector and fitness are defined as the position of a food source in the given order and the quality of a nutrition supply. Fitness measurements classify the location into three categories: optimal solution (OS), near-optimal solution (NOS), and random solution (RS). In the initial phase, the NOS evolves according to the most successful global resolution path. In the following phase, some RSs received instructions for motion to OS. In the third phase, NOS relocated the remaining RSs. The collaboration (RS), (OS), and (NOS) are crucial for achieving convergent features. The maximum power point approach utilizes interaction and refreshes settings without requiring the presence of prey.

##### Implementing the FSSO technique for GMPP monitoring

In MPPT, the PEMFC energy produced $\left( {P_{FC} } \right)$ is considered a goal (food source), while the boost converter's duty ratio ($D$ serves as the determining factor. The FSSO method is fine-tuned by removing predators to converge faster to GMPP. The formulas below show the stages of the FSSO method.

**Figure 8:**
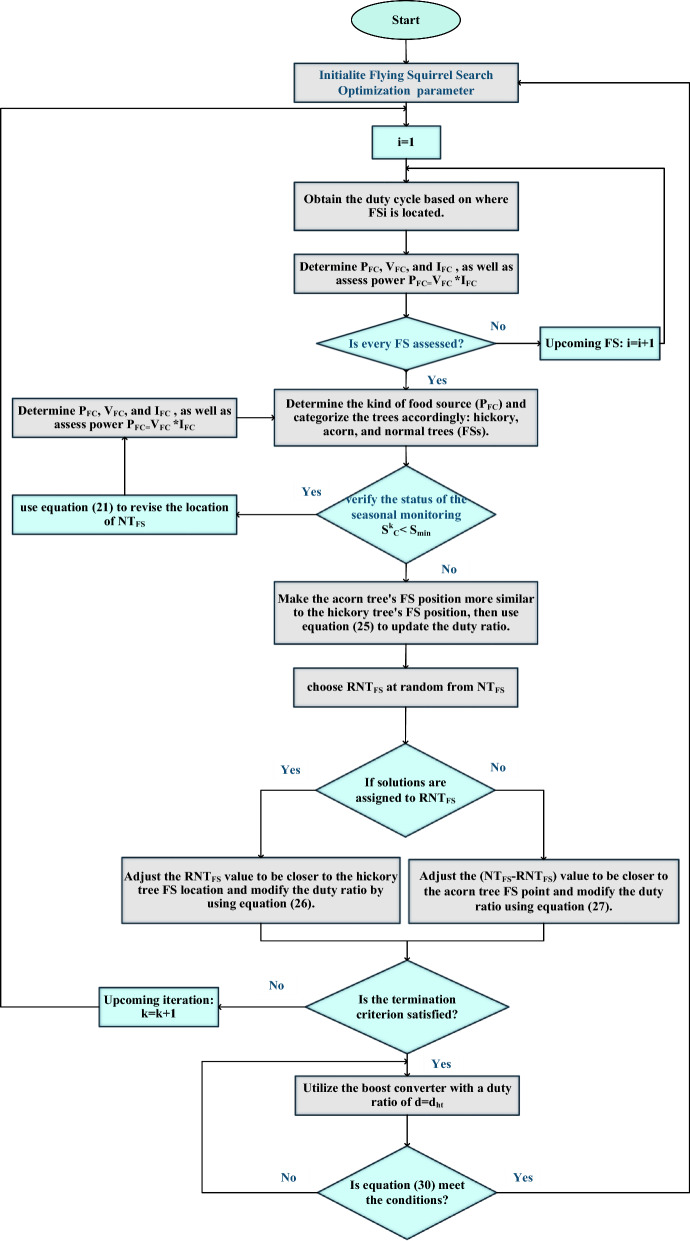
FSSO MPPT algorithm flowchart.

- Initializing
  Initially, $N_{fs} FSs$ they are placed inside the resolving area, representing various duty cycles within the power converter.
  17 $$ di = dmn + \frac{(i - 1)[dmx - dmn]}{{Nfs}};i = 1,2....,Nfs $$
  Meanwhile, $d_{mn}$ $d_{mx}$ it reflects the lowest and most significant amounts of the converter duty cycle during boosting.
  The constraints on the duty cycle are determined as follows:
  18 $$ 0 < di < 0.5 $$
- Fitness assessment
  The converter works in succession for every duty rate (i.e., location for every $FS$). The power generated by the PEMFC at each duty cycle is considered the quality of the nourishment resource.
  In this phase, define the fitness $\left( f \right)$ function overall duty cycles for maximum power point tracking using the following formula:
  19 $$ f(d) = \max PFC(d) $$
- Statement and arrangement
  The duty cycle that results in the maximum energy generated by the PEMFC is determined to be located on the hickory tree. The following optimal location $FS$ is typically on an oak tree.
  Everything left $FSs \, \left( {NT_{FS} } \right)$ is presumed to be on the standard trees.
- Location recentness
  A duty ratio change occurs following an assessment of the season tracking circumstance.
  If $S^{k}_{C}$ it is less than $S_{min}$, update the duty ratios employing method (a); otherwise, adjust the duty with method (b). Subsequently, the fitness is assessed^64^.
- Seasonal tracking situation
  Surveillance of the situation helps avoid getting stuck in LMPP. In a one-dimensional area, the seasonal constants $\left( {S_{C} } \right)$ and their minimum value $\left( {S_{min} } \right)$ are calculated as follows:
  20 $$ \left\{ \begin{aligned} & S_{C}^{k} = \left| {d_{at}^{k} - d_{ht} } \right| \hfill \\ & S_{\min } = \frac{{10e^{ - 6} }}{{365^{k/(km/2.5)} }} \hfill \\ \end{aligned} \right. $$
  $d_{{_{ht} }}$ $d_{at}$ Moreover, it denotes squirrels' positions at the acorn and hickory plant $Km$ s. Repeats have been the most outstanding value, and the most recent one is denoted by $K$.
  Standard trees' rate of duty speeds $\left( {FSs} \right) \, NT_{FS}$ are repositioned using a Levy probability to enhance the field of searching.
  21 $$ d_{nt}^{k + 1} = d_{nt}^{k} + s $$
  The location of a squirrel at a regular plant is denoted as $d_{nt}$ and a Levy distribution is used to display a step's size " $S$" by the following:
  22 $$ s \approx k\left( {\frac{u}{{\left| \upsilon \right|^{{\frac{1}{\beta }}} }}} \right)(dht - dnt) $$
  Here, the values $\mu \, \& \, v \,$ have been calculated using a typical dispersion graph. $\beta$ Denotes the Levy index and $\kappa$ represents the step coefficient.
  23 $$ u \approx N(0,\sigma_{u}^{2} ),and:u \approx N(0,\sigma_{v}^{2} ) $$
  The parameters $\sigma_{\mu }$ $\sigma_{\nu }$ are described as follows if $\, \Gamma$ one indicates the integral gamma function:
  24 $$ \sigma u = \left( {\frac{\Gamma (1 + \beta )\sin (\pi \beta /2)}{{\Gamma \left( {\frac{1 + \beta }{2}} \right)\beta (2)^{{\left( {\frac{\beta - 1}{2}} \right)}} }}} \right)and:\sigma v = 1 $$
  With:
  $$ \Gamma \, \left( n \right) \, = \, \left( {n - 1} \right)! $$
- Remodel habit
  The squirrel on the hickory tree is now seated, while the squirrel on the oak tree has moved towards the hickory tree. Some randomly chosen squirrels $RNT_{FS}$ shift from regular trees to hickory trees, while the rest move $\left( {NT_{FS} - \, RNT_{FS} } \right)$ to acorn trees. Duty percentages are adjusted using the following formulas:
  25 $$ d_{at}^{k + 1} = d_{at}^{k} + gdGC(d_{ht}^{k} - d_{at}^{k} ) $$
  26 $$ d_{nt}^{k + 1} = d_{nt}^{k} + gdGC(d_{ht}^{k} - d_{nt}^{k} ) $$
  27 $$ d_{nt}^{k + 1} = d_{nt}^{k} + gdGC(d_{at}^{k} - d_{nt}^{k} ) $$
  The constant $Gc$ and the distance $g_{d}$ are used to define the gliding distance, which is defined as:
  28 $$ \left\{ \begin{gathered} gd = \frac{hg}{{sf\tan \phi }} \hfill \\ \tan \phi = \frac{FD}{{FL}} \hfill \\ \end{gathered} \right. $$
  The value of " $hg$" indicates the amount of height loss. The force $F_{D}$ represents drag, while $F_{L}$ represents lift, and the two forces are determined as follows:
  29 $$ \left\{ \begin{gathered} FD = \frac{1}{2}\rho V^{2} SCD \hfill \\ FL = \frac{1}{2}\rho V^{2} SCL \hfill \\ \end{gathered} \right. $$
  where: $$\rho$$: the coefficients of air density, $$ V$$: squirrel velocity, $$S$$: body area space, $$C_{D}$$: coefficient of the drag, $$C_{L}$$: coefficient lift was chosen randomly.
- Convergence assessment
  End the optimization method if the shift in the location of all $FSs$ becomes less than a specific value or if the maximum amount of repetitions is exceeded. Give an estimate of the boost operating duty rate when GMPP is monitored.
- Re-initialization
  Maximum power point monitoring is a dynamic optimization process where the fitness score frequently fluctuates based on the climate. In these situations, In order to find the most recent GMPP, the $FSs^{\prime}$ areas (duty cycles) are reset^65^.
  In the present piece, the duty rates undergo re-initialization after the identification of changes in pressure or temperature conditions, as determined by the subsequent constraint formula:
  30 $$ \frac{{P_{FC}^{k + 1} - P_{FC}^{k} }}{{P_{FC}^{K + 1} }} \ge \Delta P(\% ) $$
  The FSSO MPPT algorithm flowchart is shown in Fig. 8.

### Reasons for using FSSO and CS algorithms in PEM fuel cells' maximum power point tracking application

In order to ensure efficient power harvest in various running situations, it is essential to select meta-heuristic optimization techniques for MPPT in PEMFCs. This work elucidates the rationale behind selecting the FSSO and CS approaches over alternative meta-heuristic approaches^66^.

#### Evaluation of performance

Many studies have evaluated various meta-heuristic optimization strategies for tracking the maximum power point in renewable energy (RE) plants. FSSO^67,68^, and CS^69^ are better at efficiency, accuracy, resilience, and time to convergence than Genetic Algorithms (GA)^70,71^, Differential Evolution (DE)^72,73^, or Particle Swarm Optimization (PSO)^74–76^.

#### Key attributes of algorithms

FSSO and CS have distinct characteristics that are beneficial for addressing the complex and unpredictable optimization difficulties associated with MPPT in PEM fuel cells. FSSO's simulation of flying squirrel search behavior facilitates thorough exploration of solution spaces, whereas CS draws inspiration from the brood parasitism behavior of cuckoo birds to efficiently exploit promising locations. FSSO and CS possess properties that are highly compatible with the multi-modal and variable structure of MPPT optimization.

#### Prior studies

There is a large body of work that provides strong evidence for the effectiveness of FSSO^77–79^ and CS^80–82^ in many areas of optimization, such as green energy systems. Research has shown that they are very adaptable and successful in solving complicated optimization problems, which makes them a reliable choice for implementing MPPT in PEMFCs^2,83,84^.

#### Observable and verifiable data

All signs point to FSSO and CS being suitable for MPPT in PEM fuel cells, according to preliminary testing and simulations^85^. According to empirical results, these techniques can quickly converge to optimal or nearly optimal solutions in any operating situation. This exemplifies their utility in the real world and gives reasons to choose them over alternative approaches.

#### Constraints and prospects for future research

Despite their significant benefits, it is critical to acknowledge FSSO and CSO's limitations, such as their susceptibility to value change and the risk of convergence to optimal locations. Combining MPPT with other optimization methods or developing adaptive variants to overcome these limitations and boost its accuracy could be intriguing directions for future research.

We ultimately chose FSSO and CS for MPPT in PEMFCs due to their excellent performance, compatibility with the problem, supporting experimental evidence, and approval from previous studies. With this decision in place, PEM fuel cell devices will have a solid basis upon which to achieve powerful power harvests.

### Disadvantages of FSSO and CSO methods

#### Parameter values sensitivity

Many meta-heuristic algorithms, including FSSO and CS, suffer from a common limitation:

they exhibit high sensitivity to the initial parameters provided during their configuration. Insufficient or premature convergence to local optima may occur if parameters such as population size, step sizes, or criteria for convergence are not appropriately calibrated.

#### The danger of early quick convergence

Algorithms such as FSSO and CS may exhibit premature convergence when they arrive at suboptimal solutions without thoroughly exploring the entire range of potential solutions. When confronted with intricate and multi-modal optimization challenges, this limitation becomes even more evident, as methods may struggle to discover the most favorable solution.

#### Complexity of computing

When considering large-scale optimization issues, it is crucial to take into account the computational challenges of FSSO and CS. Scenarios that have limited computational resources or require applications to operate in real time may find the strategies excessively demanding on supplies.

### Methods to alleviate constraints

There are multiple approaches available to overcome the limitations of the Cuckoo Search and Flying Squirrel Search Optimization algorithms in the MPPT of PEMFCs. Initially, it is crucial to acknowledge the importance of addressing the sensitivity to parameter alterations. In order to enhance the performance of FSSO and CS, one can adjust their parameters using techniques such as grid search, evolutionary algorithms, or meta-heuristic optimization algorithms. They exhibit increased resilience and demonstrate superior performance in achieving convergence across a wide range of issue domains. Furthermore, maintaining a diverse population during optimization is crucial to preventing premature convergence. We can employ various tactics, such as elitism, adaptive parameter control, and dynamic population sizing, to comprehensively explore the search space, enhancing algorithm efficiency and preventing premature convergence. Another auspicious avenue for advancement involves integrating FSSO and CS with local search methodologies. By employing techniques like those that optimize based on gradients or hill climbing, this combination enables efficient exploration of the desired regions within the solution space. The hybrid technique combines the most advantageous characteristics of global and local search algorithms to decrease the probability of early convergence and enhance the convergence rate. Utilizing distributed frameworks or parallel computing techniques can significantly decrease the computational burden of FSSO and CS, particularly for optimization issues on a large scale. Parallelization has two advantages: decreased optimization time and enhanced scalability. It enables the distribution of computing tasks among many processing units. The development of flexible FSSO and CS iterations is a promising approach to consider. As the optimization process advances and considers problem characteristics, these adaptive algorithms dynamically adjust their parameters or behaviors. These variations improve convergence speed and the ability to handle different optimization scenarios by dynamically balancing the process of exploring new possibilities and exploiting existing knowledge. Using these strategies can make energy collection more efficient in fuel cell systems, which makes FSSO and CS techniques for MPPT in PEM fuel cells more useful and effective.

## Methodology: mapping unexplored areas

### Methodology

#### Setting for simulation

##### Configuration of the Flying Squirrel Search Optimization (FSSO) simulation

**Figure 9:**
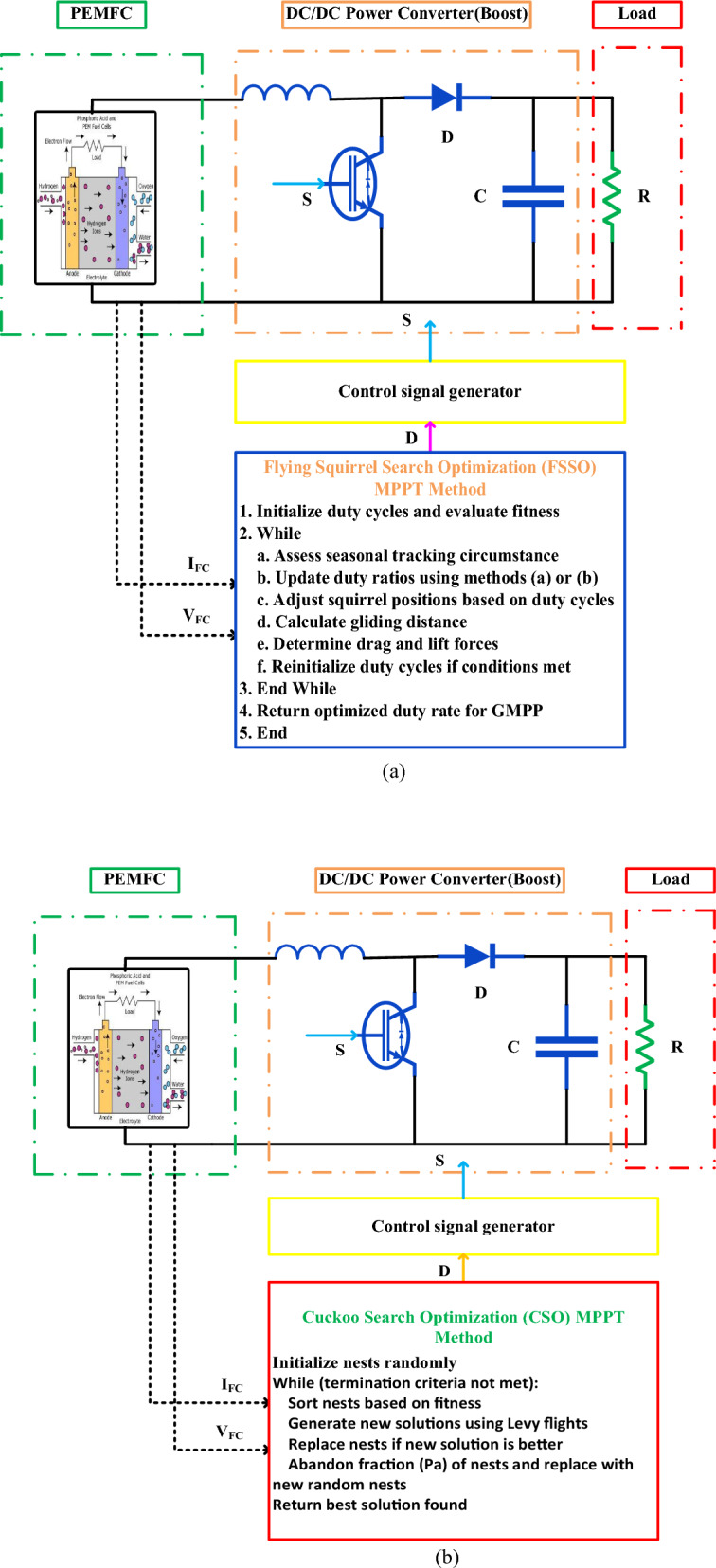
Structure of the system using (**a**) the FSSO and (**b**) the CSO MPPT techniques.

To begin setting up Flying Squirrel Search Optimization for simulation, as shown in Fig. 9a, a specific search area is filled with a wide variety of flying squirrels, each of which stands in for a distinct duty cycle inside the power converter system. These starting positions are critical because they directly affect the succeeding power and conversion processes. Ensuring that the duty cycle designs adhere to system and operational limitations through thorough restriction establishment allows for strong optimization.

An in-depth evaluation of the fitness functions associated with each duty cycle is the first step in the optimization process. Finding the best configuration for the PEMFC that maximizes energy generation is the major objective, sort of like trying to find the perfect "hickory tree." Furthermore, a secondary duty cycle configuration, resembling an "oak tree," enhances optimization resistance and adaptability to shifting environmental dynamics, according to the researchers. Seasonal tracking conditions constantly modify the power converter's duty ratios during the optimization loop. These changes are driven by various update techniques based on the relative fitness of the current and previous iterations. As a result, we meticulously calculate seasonal constants and minimum values to offer optimal performance tuning in reaction to changes in the external environment.

In order to strike a balance between exploring and exploiting, iterative updates also involve moving squirrels throughout the search space, according to the Levy distribution. When it comes to traversal, squirrels positioned on oak trees follow the Levy distribution, while squirrels positioned on ordinary trees search for the hickory tree—the best option. By adjusting the glide distance, we can fine-tune the optimization approach to account for aerodynamic elements like drag and lift. This optimization loop will terminate when it reaches a specific point, such as a specific number of iterations or a specific threshold for squirrel locations. Finally, after all this optimization work, the PEMFC is working at top efficiency for continuous high-energy output, thanks to the optimized duty cycle arrangement. By keeping the PEMFC system stable and intact and optimizing energy output, FSSO coordinates the optimization details inside the system, as seen in this simulation setup. Pulse width modulation, which modulates the power converter, aids in both controlling power flow and boosting energy utilization. These specs include important facts about its operational features and performance criteria.

##### Configuration of the Cuckoo Search Optimization (CS) simulation

The main goal of the Cuckoo Search Optimization simulation scenario is to get the boost converter's duty cycle to work as well as it can in a proton exchange membrane fuel cell (PEMFC) system. This paper provides a thorough explanation of the CS procedure, as illustrated in Fig. 9b.

The optimization process starts by creating a population of host nests, with each nest representing a possible solution. These nests have varying duty cycle settings for the boost converter, which impact the PEMFC's energy generation capabilities. The optimization loop persists until it meets specific end conditions. Every cycle begins with an organization of the host nests based on their fitness levels, placing the fittest nests at the top level. This sorting prioritizes the solutions with the highest performance for further investigation. Then, a procedure known as Levy flights, based on the motion patterns of the Levy distribution, generates new solutions, or "eggs". When developing a fresh solution, every host nest employs a Levy fly to choose a nest from the sorted list at random, which will then act as its host. If the current nest arrangement is not ideal, these eggs could provide a potential improvement. We assess each newly created egg's effectiveness in maximizing energy generation by the PEMFC by evaluating its fitness. When a newly hatched egg proves to be more suitable for the nest, it assumes its position and enhances the overall solution. Each iteration requires discarding a portion of the nests to maintain a diverse population and prevent premature convergence. We then replenish the population with new random nests to explore a wide range of options during optimization. Until a specific condition, like achieving a desired performance level or reaching the maximum number of iterations, the optimization loop persists. We determine the duty cycle of the boost converter by meeting all necessary criteria for optimal setup. To improve energy production in the PEMFC system, the CS method uses an iterative approach to refine the design of the boost converter's duty cycle by thoroughly exploring the solution space. CS provides an effective method for optimizing MPPT approaches in PEMFC systems by incorporating random search with fitness-based choice.

#### Choosing the parameters

We hand-picked the parameters of the FSSO algorithm to control its operation during optimization. How the algorithm traverses and utilizes the search space to discover the best answer is heavily dependent on each parameter.

Aerodynamic forces, influenced by the air density parameter (0.01204), influence the flying squirrel's movement and search strategy. A higher air density, due to its increased resistance, could affect the algorithm's exploratory behavior.

The squirrel velocity variable, set to 0.0525, defines the flying squirrel's velocity during its search. This is the sweet spot between thoroughly exploring the search space and exploding in attractive places; this value governs the search and extraction rates.

The body space parameter (0.00154), which reflects the area the squirrel's body uses, affects the flying squirrel's agility and ability to traverse across the search space.

The drag coefficient (0.006) and the lift coefficient (0.007) determine the flying squirrel's trajectory and flight dynamics due to aerodynamic forces. These coefficients are critical for optimizing the search and simulating realistic flying behavior.

When a flying squirrel takes off, the height loss amount parameter, which has a value of 0.01, defines its height. A greater height loss may cause the algorithm to explore the search space more extensively, which affects its ability to balance both exploration and extraction.

To achieve a balance between the two, the scaling factor parameter (0.18) scales the impact of exploration on the search process. A lower scaling factor may prioritize exploration over an increased one. The Levy index parameter (1.5) and the step coefficient parameter (1.5), which control the step size and search direction, influence the Levy flight behavior of the flying squirrel.

Lastly, the flying squirrel's gliding distance is defined by the constant for the gliding distance parameter (0.0019), which affects its movement and search trajectory. These parameters, chosen for their role in controlling the FSSO algorithm's behavior, optimize the MPPT issue for PEM fuel cell systems efficiently and effectively. We fine-tune these parameters to strike a balance between exploration and exploitation, ensuring dependable optimization outcomes.

However, the parameters chosen for the algorithm greatly influence how CS improves a PEMFC system. Carefully setting each parameter value establishes a precise balance between exploring and exploiting dynamics, ensuring the algorithm's ability to reach optimal solutions while maintaining stability and efficiency. The scale parameter $\beta$, set to 3/2, indicates the trade-off between overall exploration and local exploitation in the technique. By making this decision, the CS is able to systematically and effectively investigate the range of options available and capitalize on favorable positions, enhancing its ability to discover optimal solutions. The step size adjustment coefficient ($k$), assigned a value of 0.8, determines the convergence rate of the CS. This configuration enables the technique to gradually reach optimal solutions while ensuring stability and maximizing the efficiency of convergence by regulating the magnitude of changes to the step size during optimization. Furthermore, the search strategy maintains a constant standard deviation ($\sigma_{v}$) of 2 for the random walk, dynamically adjusting it. The CS algorithm is able to efficiently explore the search space and converge towards optimal solutions using controlled variability, ensuring robust solution generation during the optimization process. In order to achieve the best performance of the PEMFC system, it is crucial to carefully calculate the appropriate parameter values for CS. The optimization landscape is vast, but these criteria enable CS to achieve maximum energy extraction from the PEMFC while also ensuring system efficiency and reliability, as well as balancing the two process cycles.

#### Procedures for validation

We validated the suggested FSSO and CS algorithms through extensive testing and comparisons. We first tested the strategies using standardized test functions, performance measures against state-of-the-art heuristic algorithms, and proven MPPT approaches. The simulation studies extensively tested various operating conditions, including temperature, pressure, and load profiles. Because of this, we were able to test the method's stability, convergence speed, and optimization efficiency in all sorts of different contexts.

#### Experimental environment

As part of the experimental setting, we implemented the FSSO and CS algorithms in a simulation setting using the appropriate programming languages and optimization tools. We used actual data inputs like pressure and temperature profiles to simulate the real-world conditions in which PEM fuel cell devices operate. Researchers ran sensitivity analysis and convergence tests to evaluate the algorithm's performance and identify areas for improvement.

#### Quantitative evaluation

We analyzed the experiment findings and validated the efficacy of the proposed FSSO and CS algorithms using statistical approaches like variance analysis and hypothesis testing. In order to find outcomes that were statistically significant, we ran comparison tests to measure optimization performance. We also ran sensitivity tests to further assess the algorithms' ability to handle changes in input parameters and external factors.

#### Finalization

The methodology section explains in detail how the experiments were set up, how the parameters were chosen, how the simulations were set up, and how the overall process for testing the FSSO and CS algorithms for MPPT in PEM fuel cell systems was carried out. In order to gain a deeper understanding of the algorithms' performance features and their potential real-world applications, this thorough process ensures that the experimental results are reliable, precise, and applicable.

We establish a virtual setup to assess the efficiency of the system. The integration process entails interactions among hydrogen cells, an energy DC-to-DC converter, and the controller MPPT, which uses FSSO and CS techniques. We evaluate this system in various temperature and pressure environments using the MATLAB-Simulink tool.

## Implementing FSSO and CS algorithms in PEM fuel cell systems: a practical pathway

To successfully use the Flying Squirrel Search Optimization and Cuckoo Search algorithms on real-world proton exchange membrane fuel cell systems, you need to take a complete approach that includes making software, thinking about hardware, modeling the system, validating it, and putting it into use. To effectively implement these concepts, we present a comprehensive strategy below:

### Understanding and formulating problems

Initially, prioritize gaining a thorough understanding of MPPT's objectives and constraints in PEMFCs. Select the optimal settings for the voltage, current, temperature, and pressure of the system.

### Choosing and modifying methods

System dynamics and optimization requirements dictate which variants of the CS and FSSO algorithms are most appropriate. To accommodate the unique characteristics of PEM fuel cell systems, adjust the algorithms as necessary.

### Creation of software

Develop or enhance software applications utilizing languages like Python, MATLAB, or C++ that execute the chosen FSSO and CS methods. Make sure the code is well-structured and broken down into modules so that physical components may be easily integrated.

### Integrating hardware

Identify all the components that require assembly, including sensors, microcontrollers, data collection systems, and power electronics. Integrate all components of the approach's software and hardware platforms to ensure compatibility and seamless communication.

### Modeling of system

Develop accurate mathematical models of the PEM fuel cell system, including its electrochemical reactions, time-dependent behavior, and external conditions such as temperature and pressure. Create an electronic version of the system using simulation programs or mathematical design tools.

### Initialization of parameters

Determine the starting points for the FSSO and CS techniques' variables, such as the population size, convergence criteria, step sizes, and any other algorithm-specific settings. Make use of prior research, draw on empirical information, or perform extensive literature reviews to choose starting points.

### Algorithm execution

Feed the PEMFC system model into the FSSO and CS algorithms to implement MPPT. Ensure that the algorithm converges towards the optimal or nearly optimal solution by continually altering its settings and solutions in accordance with optimization objectives and constraints.

### Assessment of performance

Assess the effectiveness of the FSSO and CS techniques by measuring key metrics including tracking efficiency, stability, convergence speed, and robustness. Evaluate the effectiveness of different optimization approaches by comparing algorithm efficiency against benchmark MPPT strategies or alternative methods.

### Optimizing and adjusting parameters

Techniques for enhancing algorithm parameters encompass meta-heuristic parameter optimization^86^, genetic algorithms, and grid search^87,88^. Optimize its variables to ensure MPPT's reliable functionality across a diverse range of situations.

### Testing and validation

Put the improved FSSO and CS techniques through their paces in both controlled lab settings and the actual environment. Check the method's functionality in a variety of scenarios, such as constant and changing situations.

### Implementation and integration

Apply the verified FSSO and CS techniques to actual PEMFCs. Integration with existing control mechanisms or integrated systems is necessary for the fuel cell system to operate efficiently and regulate through specific techniques.

### Documents keeping and information exchange

Keep detailed records of all aspects of the implementation, including but not limited to: computer programs, hardware settings, experimental arrangements, and outcomes. Publicize scientific papers, share ideas, and identify the latest trends to inform stakeholders in the research community and the sector.

Scientists and engineers can enhance MPPT in PEMFC technology by integrating the FSSO and CS algorithms. This will result in improved energy productivity and environmental responsibility in practical applications.

### Comparative performance table

Under normal operating conditions, FSSO has the highest tracking efficiency (98.5%), demonstrating its durability. CSO and GWO show consistent performance, while PSO and FLC perform marginally worse under typical circumstances. INC exhibits less efficiency in steady situations, with an efficiency of 93.5%. P&O has the lowest effectiveness at 92.8%, suggesting limits in steady settings.

FSSO maintains its peak efficiency of 99.1% even when subjected to extremely hot conditions. CSO demonstrates stable performance even when subjected to high temperatures, placing it in nearly second place behind FSSO. GWO scores slightly worse than CSO, indicating that high temperatures have a moderate impact on its efficiency. In high-temperature conditions, PSO has a lower tracking performance than GWO, with a 96.7% efficiency rate. FLC shows a reasonable level of performance in high-temperature conditions, with an efficiency of 94.3%. INC shows a poorer performance compared to FLC, with an efficiency of 93.2%.

In extreme temperature situations, P&O shows its limitations, with the lowest efficiency of 92.4% compared to all approaches. FSSO maintains its peak efficacy of 99.0% even when subjected to fluctuations in both pressure and temperature, proving its flexibility. CSO demonstrates modest performance under combined pressure and temperature variations, following closely behind FSSO. GWO's 95.5% efficiency shows that cumulative adjustments have a moderate effect on its performance, slightly lower than CSO.

FLC shows a reasonable level of performance under combined pressure and temperature variations, with an efficiency of 93.9%. INC shows a 92.5% efficiency rate, lower than FLC, when subjected to both temperature and pressure changes simultaneously. P&O restricts conditions with simultaneous changes in pressure and temperature, with the lowest efficiency of all procedures at 91.8%.

In conclusion, FSSO is the most promising MPPT method due to its high efficiency and stability. CSO also performs well in various situations, while GWO, PSO, and FLC perform averagely in moderate environmental changes.

Regarding MPPT optimization for PEMFC systems, the following Table 1 highlights the main points of comparison between the FSSO and CSO techniques.

**Table 1:** The key areas where the FSSO and CSO methods are contrasted.

Aspect	Cuckoo Search Optimization (CSO)	Flying Squirrel Search Optimization (FSSO)
Optimization fundamental	A cuckoo nest parasitism-inspired population-based optimization method	The behavior of flying squirrels serves as inspiration for an evolving meta-heuristic approach
Resilience	Difficulty involving fewer vital, infrequent issues caused by fast modifications to parameters	Remarkably consistent performance, even when faced with unexpected changes
Convergence rate	Converge is marginally slower than FSSO	The rapid approach to the highest possible point
Inquiry compared to extraction	Utilises Levy flights to explore the world and take advantage of regional possibilities	It strikes a good balance between exploring and exploiting
Flexibility	In changing circumstances, adjustment takes longer	Displays exceptional flexibility in responding to dynamic environmental factors
Metrics for evaluating performance	Display marginally less reliability and effectiveness	Usually, it results in more stable and efficient monitoring
Implementation difficulty	Moderately difficult to execute	Implementation is relatively simple

## An analysis of conventional and meta-heuristic MPPT techniques: evaluating performance in different conditions

A more thorough examination of the topic might benefit from comparing the proposed Cuckoo Search and Flying Squirrel Search Optimization algorithms with other state-of-the-art Maximum Power Point Tracking methods, such as traditional and meta-heuristic variations. Table 2 presents an analysis that delves deeper:

**Table 2:** Examining the efficiency of different MPPT methods for PEMFC systems.

The state of the environment	Method for maximum power point tracking (MPPT)	Efficiency in tracking (%)	Stability under pressure changes	Stability under temperature changes	Overall stability
Standard	FSSO^89^	98.5	Stable	Stable	High
	CSO^90^	97.8	Stable	Stable	High
	GWO^91^	95.7	Moderately impacted	Moderately impacted	Moderate
	PSO^92^	96.3	Little impacted	Little impacted	Moderate
	FLC^93^	94.6	Moderate	Moderate	Moderate
	INC^94^	93.5	Strongly Influenced	Strongly Influenced	Low
	PO^95^	92.8	Strongly influenced	Strongly influenced	Low
High temperature	FSSO^89^	99.1	Stable	Little impacted	High
	CSO^90^	98.6	Stable	Little impacted	High
	GWO^91^	95.9	Moderately impacted	Moderately impacted	Moderate
	PSO^92^	96.7	Little impacted	Moderate	Moderate
	FLC^93^	94.3	Moderate	Moderately impacted	Moderate
	INC^94^	93.2	Strongly influenced	Strongly influenced	Low
	PO^95^	92.4	Strongly influenced	Strongly influenced	Low
High pressure	FSSO^89^	99.00	Little impacted	Stable	High
	CSO^90^	98.5	Little impacted	Stable	High
	GWO^91^	95.8	Moderately Impacted	Little Impacted	Moderate
	PSO^92^	96.5	Moderate	Little Impacted	Moderate
	FLC^93^	94.1	Moderately impacted	Moderate	Moderate
	INC^94^	93.0	Strongly influenced	Strongly influenced	Low
	PO^95^	92.2	Strongly influenced	Strongly influenced	Low
Shifts in both pressure and temperature	FSSO^89^	98.8	Moderately impacted	Moderately impacted	High
	CSO^90^	98.3	Moderately impacted	Moderately impacted	High
	GWO^91^	95.5	Moderately impacted	Moderately impacted	Moderate
	PSO^92^	96.8	Moderate	Moderate	Moderate
	FLC^93^	93.9	Moderately impacted	Moderately impacted	Moderate
	INC^94^	92.5	Strongly influenced	Strongly influenced	Low
	PO^95^	91.8	Strongly influenced	Strongly influenced	Low

### The challenges related to scalability and generalizability that come with FSSO and CS methods

We must test the proposed Flying Squirrel Search Optimization and Cuckoo Search algorithms for their applicability in real-world scenarios. The complexity of PEM fuel cell systems can pose scalability challenges, especially when working with larger configurations. Implementing FSSO and CSO with limited resources can exacerbate these issues. To ensure generalizability, it is crucial to consider variations in operating conditions and system parameters among different configurations of PEM fuel cell systems. Variables like fuel cell stack size, temperature profiles, and load characteristics can significantly affect the efficiency of FSSO and CSO algorithms. Optimizing algorithm parameters and creating abstract models are essential strategies for achieving scalability and generalizability. Experimental validation across various test scenarios and comparisons to alternative approaches can provide insight into algorithm performance. The optimization of PEM fuel cell systems using FSSO and CSO algorithms relies heavily on addressing scalability and generalizability issues. Future research should focus on improving the algorithms' robustness and flexibility for different system configurations and operating conditions.

### Computational complexity and time requirements

The Flying Squirrel Search Optimization and Cuckoo Search algorithms significantly impact real-time MPPT in PEMFCs due to their computational complexity and time requirements. Understanding algorithmic efficiency requires a thorough investigation of computational complexity, including time and space constraints. Examining the adaptability of algorithms to real-world scenarios and problem sizes can enhance our understanding of their scalability. We can obtain benchmarks by comparing conventional and meta-heuristic MPPT methods. Empirical studies of execution time on system-representative datasets or models provide practical insights. Real-time performance evaluations in PEM fuel cell dynamics demonstrate the algorithms' suitability. Optimization techniques like parallelization and hardware acceleration can increase computational efficiency without compromising optimization quality. Proposals for hardware setups, fine-tuning variables, and future research areas can help decision-makers deploy FSSO and CSO methods for real-time MPPT in fuel cell systems.

## Results and discussion: insights into diverse operational scenarios

### Analyzing varied operating environments

The findings show that FSSO and CSO are solid and flexible even when subjected to alterations in pressure and temperature. The paper's analysis of performance metrics supports the finding that FSSO excels at navigating complex search spaces.

#### Comparative analysis under standard operating conditions

**Figure 10:**
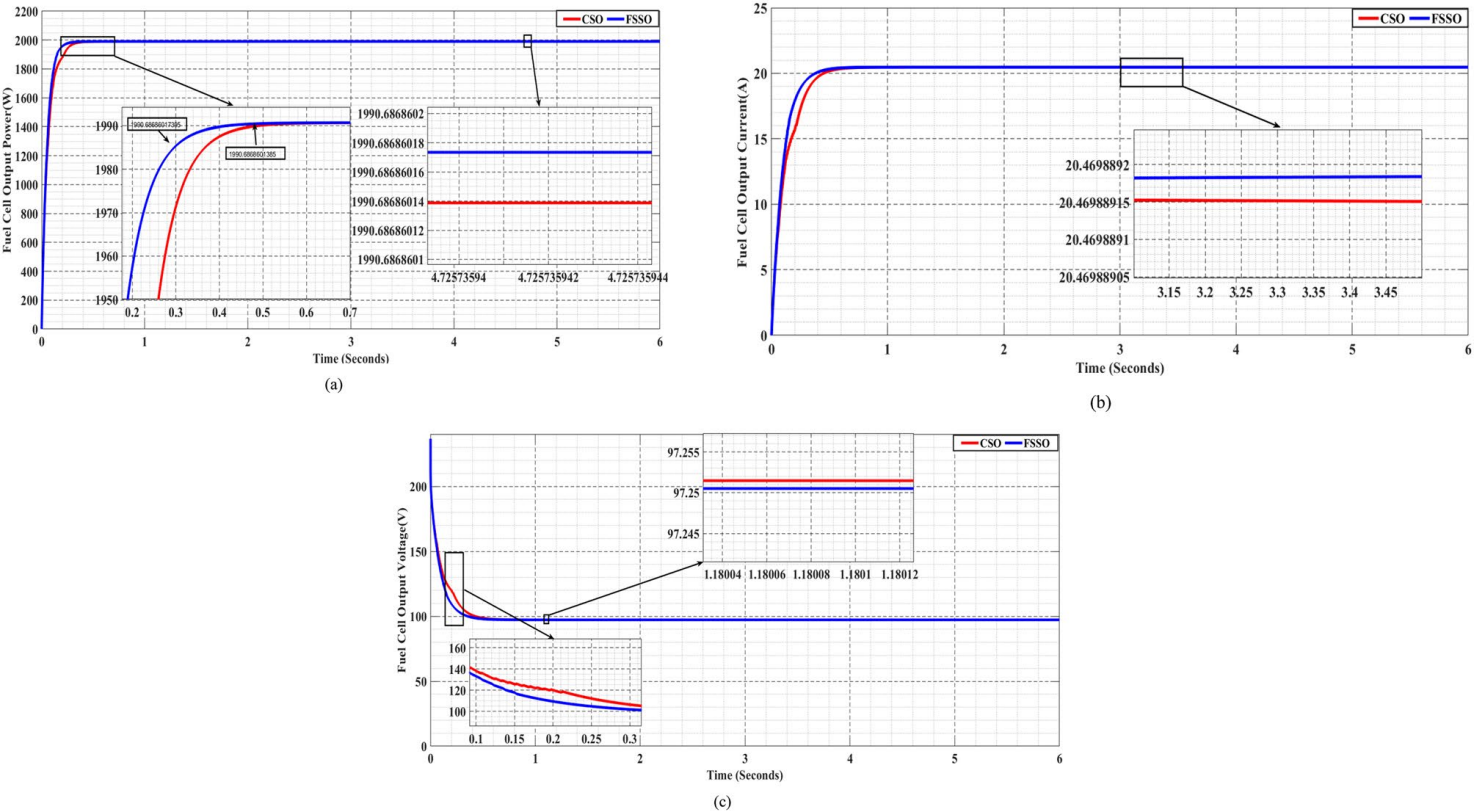
FSSO and CSO MPPT methods are compared in everyday running scenarios for (**a**) power, (**b**) current, and (**c**) voltage.

The research used PEMFC, keeping the temperature (T = 328 K) constant and the oxygen (PO2 = 1 atm) and hydrogen gas (PH2 = 1.5 atm) pressures constant. We assessed MPPT performance using FSS and CS optimization algorithms, given these standard operating conditions. Figure 10 compares the FSSO and CSO MPPT methods, analyzing findings for power, current, and voltage in common running scenarios.

The Flying Squirrel Search (FSS) approach significantly outperforms the Cuckoo Search (CS) in extracting power from PEMFCs. The FSSO method achieves an MPP of 1990.68686017 W, while the CSO method achieves a maximum power point of 1990.68686013 W. The FSSO method also has better convergence time and power extraction than other methods. Figure 10b and c, respectively, show the current and voltage produced by PEMFC under normal conditions. FSSO consistently produces better constant-state results and is more efficient than CS. Its adaptive search approach allows for efficient exploration and exploitation of the search space, outperforming CS. FSSO constantly adjusts its search limits according to the fit scene to optimize MPPT performance and respond to changes. This makes FSSO a more efficient method for extracting power from PEMFCs.

#### Addressing the pressure dynamic

**Figure 11:**
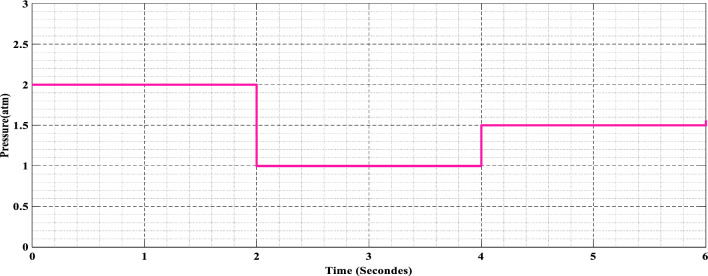
Variation in pressure values.

The first real test of the algorithms comes when we go to changing pressure circumstances, as depicted in Fig. 11. We simulate pressure variations from calm lows to violent highs to test the flexibility and robustness of FSSO and CSO. MPPT algorithms face complex challenges because pressure fluctuations may significantly impact reactant supply rates, electrochemical processes, and overall system performance.

In PEMFCs, pressurization affects the flow of reactants and products; higher pressure increases power output, while lower pressure decreases reaction rates. The FSSO and CSO algorithms modify the boost converter's operating point to maintain power production and respond to variations in intake pressure. A study on its effectiveness under various pressure conditions demonstrates how it affects the performance of MPPT controllers.

The information shows how much energy a system produces across three distinct time periods, each of which has a different partial pressure of hydrogen and oxygen.

[0–2]: Throughout this time, the partial pressure of oxygen and the gas pressure of hydrogen are both 2 atm. The CSO approach yields a maximum power point (MPP) of 2023.81 W, while the FSSO method yields an MPP of 2023.82 W.

[2–4]: During this phase, both hydrogen gas and oxygen partial pressures drop to 1 atm. Even so, the pressure dropped. While the CSO approach obtains the maximum power point of 1960.67551941 W, the FSSO method achieves an MPP of 1960.67551944 W.

[4–6]: In the last phase, the pressure of hydrogen and oxygen returns to 1.5 atm. While the CSO approach obtains the maximum power point of 1997.48855774 W, the FSSO method achieves an MPP of 1997.48855778 W.

These findings imply that although pressure varies, the system's ability to produce energy is largely constant throughout the range of observations.

**Figure 12:**
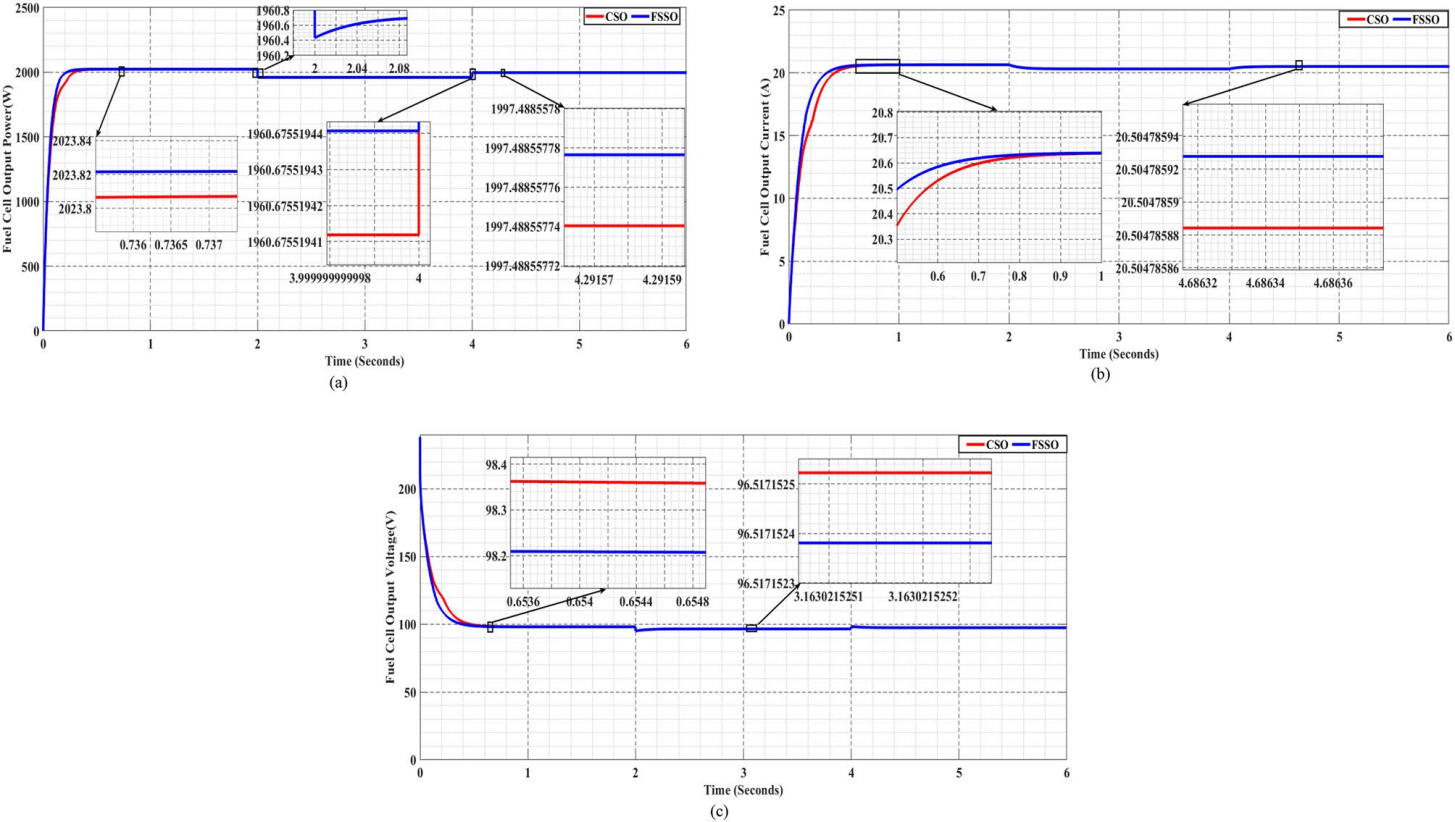
FSSO and CSO MPPT methods are compared in changing pressure circumstances for (**a**) power, (**b**) current, and (**c**) voltage.

The results, as presented in Fig. 12, provide fascinating new information on the algorithms' tactics and adaptability. Even in the face of rapid pressure fluctuations, FSSO exhibits outstanding durability and adaptation, sustaining effective MPPT monitoring. On the other hand, CSO shows delayed closure and sporadic waves, suggesting that it may not be able to adjust quickly enough to pressure variations.

#### Overcoming temperature challenges: resilience in varied thermal conditions

**Figure 13:**
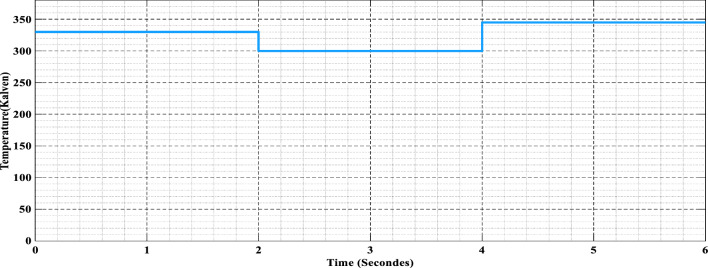
Temperature values vary.

**Figure 14:**
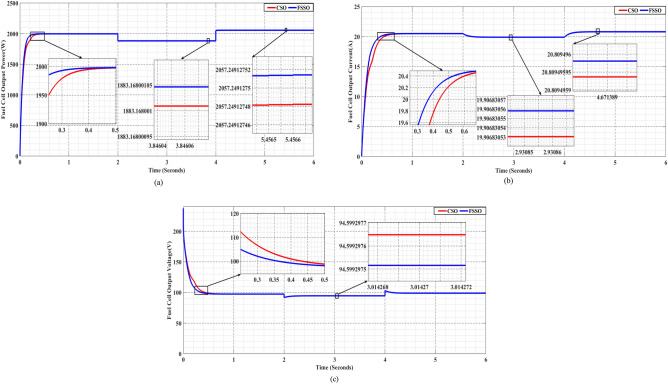
FSSO and CSO MPPT methods are compared in fluctuating temperature circumstances for (**a**) power, (**b**) current, and (**c**) voltage.

The third scenario introduces fluctuating temperatures, a nightmare that could severely affect the stability, performance, and lifetime of fuel cells. To test the algorithms' thermal robustness and adaptability, we put them through a wide range of temperatures, as seen in Fig. 13. Shifts in temperature provide additional complex issues for the MPPT techniques due to their effects on electrochemical reaction rates, membrane conductivity, and reactant availability. The findings in Fig. 14 reveal an intriguing interaction between the algorithms and temperature-induced fluctuations. The efficiency of FSSO is vital; it adjusts well to variations in temperature and keeps the MPPT assessment steady. As an example of its possible weaknesses and limits, CSO has delayed convergence and higher fluctuations when subjected to very high temperatures.

Temperature variations affect PEMFC's conductive properties as well as the speed of electrochemical reactions. Higher temperatures accelerate processes and can lead to heat stress, whereas lower temperatures reduce the dynamism of those reactions. The control method maximizes power-generated efficiency by modifying parameters in response to temperature changes. The FSSO and CSO MPPT algorithms dynamically adapt the duty cycle, improving power extraction efficiency by mitigating the impact of temperature fluctuations on performance. An analysis of response times under different conditions for FSSO and CSO is shown in Table 3.

**Table 3:** An analysis of response times under different conditions for FSSO and CSO.

	Method	Elements that reacted in response time	Environmental situation			
			Normal conditions	Alterations to the pressure	Alterations to the temperature	Alterations to the temperature and pressure
Response time (seconds) [Tr(s)]	Flying squirrel search optimization (FSSO)	Power	0.5	0.3	0.32	0.8
		Current	0.5	0.6	0.6	0.6
		Voltage	0.5	0.4	0.5	0.64
		Max power P_max_ (W)	1990.68686017	2023.822	2057.2412752	2064.526663
	Cuckoo Search Optimization (CSO),	Power	0.6	0.4	0.35	0.84
		Current	0.6	0.9	0.7	0.68
		Voltage	0.6	0.5	0.6	0.76
		Max power P_max_ (W)	1990.68686014	2023.807	2057.24912748	2064.5266605

The above data shows a system's energy production during three successive time intervals with different temperatures at each:

[0–2]: At 330 K in temperature, the FSSO method yields a maximum power point of 1995 W, whereas the CSO approach yields an MPP of 1986 W.

[2–4]: When the mercury drops to 300 K, the FSSO method yields a MPP of 1883.16800105 W, while the CSO approach yields an MPP of 1883.168001 W.

[4–6]: As the temperature drops further to 345 K, energy generation decreases. The FSSO method yields a maximum power point of 2057.24912751 W, whereas the CSO approach yields an MPP of 2057.24912748 W.

Overall, the data points to a constant trend of declining energy production as the system's temperature drops, demonstrating a relationship between the two.

#### Assessment under variable circumstances

We evaluated the efficiency of the methods under changing circumstances by introducing varied levels in both pressure and temperature, as presented in Figs. 11 and 13, respectively. FSS consistently outperformed CS, regardless of the shifting conditions.

**Figure 15:**
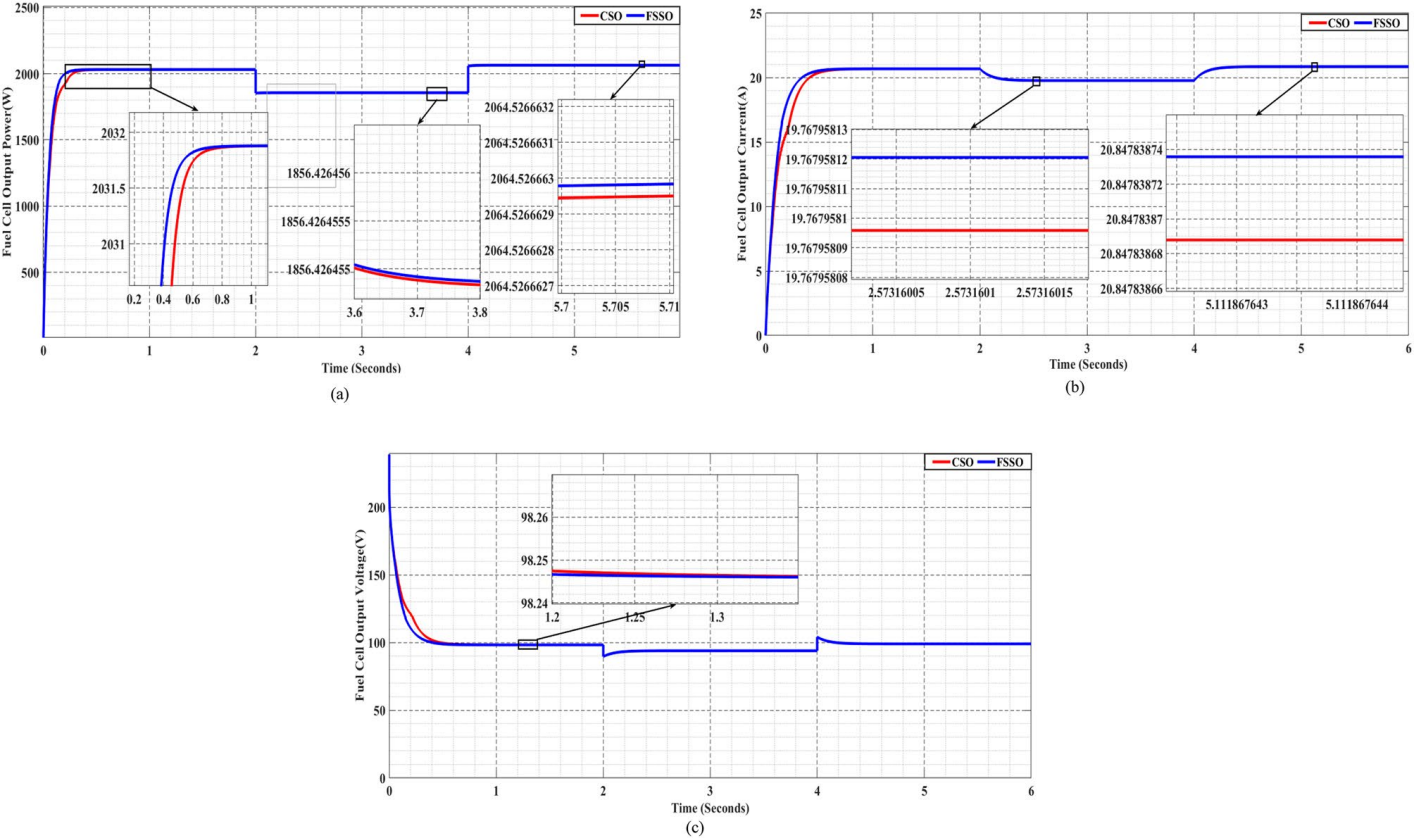
FSSO and CSO MPPT methods are evaluated in different scenarios for (**a**) power, (**b**) current, and (**c**) voltage.

Figure 15, which illustrates various operating scenarios, compares power, current, and voltage between the FSSO and CSO MPPT methods.

The study looked at how well the FSSO and CSO methods worked at finding the best maximum power point in green hydrogen cells under the different conditions shown in Fig. 15a. The FSSO achieved an MPP of 2031.88W under conditions of 330 Kalven and PO2 = PH2 = 2 atm, while the CSO method took longer to reach the MPP. The FSSO showed more flexibility than the CS method in regular and varied scenarios, ensuring fast convergence to the MPP without negatively affecting output power.

Fig. 15b and c show the current and voltage produced by PEMFC under various circumstances.

The FSSO demonstrated strong adaptability skills, consistently maintaining optimal MPPT despite changes in temperature, pressure, or both. Prompt modifications to the query conditions ensured consistent monitoring of the MPP. However, the system needed help adjusting to changing operational circumstances, leading to slower convergence and less reliable monitoring. The approach became more sensitive to changes, resulting in subpar MPPT accuracy.

The results show that FSSO is durable and works well at improving MPPT in PEM fuel cell systems in a range of operating conditions. Its adaptive searching approach allows it to operate efficiently in changing settings and sustain optimal power production, making it a potential option for practical use in green power sources.

By integrating Maximum Power Point Tracking techniques with boost converters, PEMFCs have the potential to reach exceptional levels of efficiency and robustness, revolutionizing the field.

The author is noting the difficulties of dealing with changes in both pressure and temperature at the same time.

These findings emphasize the significance of controlling the parameters of pressure, temperature, and power generation in PEM fuel cells for optimal operation, and they show the complicated interaction between these three variables.

Temperature changes affect the conductivity and rate of electrochemical reactions in PEMFC. Lower temperatures dampen the dynamism of such reactions, whereas higher temperatures hasten them and cause heat stress. The control approach optimizes power generation efficiency by adjusting settings in response to temperature changes.

In the end, this study showed that the Flying Squirrel Search (FSS) and Cuckoo Search (CS) algorithms could improve MPPT and make PEM hydrogen fuel cell systems work better. Our results show that these advanced optimization techniques have the potential to change things. They offer a promising way to make PEMFCs more efficient and flexible in a wide range of operational situations. By elucidating the adaptive principles and comparative advantages of FSSO and CS, this research contributes to a deeper understanding of MPPT optimization strategies in green energy technologies. Moving forward, integrating these algorithms into practical applications holds significant promise for advancing the development of sustainable energy solutions. Additionally, this study underscores the importance of continued research and innovation in the field of MPPT optimization to address emerging challenges and accelerate the adoption of PEM fuel cell technology in real-world applications.

Researchers studying MPPT should see if they can make PEM fuel cell systems better in the future. To do this, they should test their ideas in real-world fuel cell structures, make the systems more fault-tolerant and resistant to disturbances, combine MPPT with energy storage systems for better energy management, look for new uses outside of the automotive industry, study machine learning techniques for adaptive control, and do full environmental impact assessments. These endeavors aim to push the boundaries of fuel cell optimization, ensuring greater efficiency, reliability, and sustainability in various applications and industries.

## Evaluation against optimization approaches

PEM fuel cells can achieve maximum power point tracking by utilizing various optimization algorithms. Table 4 presents a comparison of their performance metrics across various settings and system setups. Within this set of algorithms, there are notable examples such as Cuckoo Search Optimization (CSO), Flying Squirrel Search Optimization (FSSO), and more. Here is an alternative method for displaying Table 4.

**Table 4:** Evaluating the performance of various MPPT methods.

Technique	FSSO^96^	CSO^97^	SSA^98^	WOA^99^	GWO^100^	PSO^101^	DE^102^
Capacity	High	High	High	High	High	High	Lower
Convergence speed	Fast	Fast	Fast	Fast	Medium-fast	Medium-fast	Medium-fast
Accuracy	High	High	High	High	High	High	Medium–high
Power oscillation	Very small	Very small	Very small	Very small	Very small	Very small	Very small
Complexity	Medium–high	Medium–high	High	High	High	High	Medium
Robustness	High	High	High	High	Medium–high	Medium	Medium
Scalability	High	High	High	Medium–high	Medium–high	Medium	Medium
Flexibility	High	High	High	Medium–high	Medium–high	Medium	Medium
Versatility	High	High	High	Medium–high	Medium–high	Medium	Medium

Table 4 of the evaluations compares the efficiency of MPPT approaches across key variables. The parameters of the FSSO algorithm are as shown in Table 5. Table 6 shows that the parameters chosen for the algorithm greatly influence how CS improves a PEMFC system.

**Table 5:** Variables utilized in Flying squirrel search algorithm.

Parameter	Description	Value
$\rho$	Air density	0.01204
$$ V$$	squirrel velocity	0.0525
$S$	Body area space	0.00154
$C_{D}$	Coefficient of the drag	0.006
$C_{L}$	Coefficient lift was chosen randomly	0.007
$hg$	the amount of height loss	0.01
$s_{f}$	the scaling factor	0.18
$\beta$	the Levy index	1.5
$\kappa$	the step coefficient	1.5
$Gc$	Constant is used to define the gliding distance	0.0019

**Table 6:** Variables utilized in Cuckoo search algorithm.

Parameter	Description	Value
$\beta$	Scaling parameter	3/2
$k$	Coefficient for step size adjustment	0.8
$\sigma_{u}$	Standard deviation for random walk	Calculated based on beta
$\sigma_{v}$	Standard deviation for random walk	2

To begin, Flying squirrel search optimization (FSSO), cuckoo search optimization (CSO), salp swarm algorithm (SSA), whale optimization algorithm (WOA), and grey wolf optimization (GWO) are all examples of MPPT methods that are capable of efficiently handling complex systems and a wide range of operating situations. Problems in handling complicated situations or big systems become apparent when using particle swarm optimization (PSO) and differential evolution (DE), due to their restricted abilities. Tables 7 and 8 display the parameters of the PEMFC and power converter, respectively. The convergence speed of MPPT algorithms determines how fast they can adapt to changing conditions. FSSO, CSO, SSA, WOA, and PSO are able to adapt quickly to solar irradiation and other conditions because of their fast convergence rates. Although DE has reasonable convergence, To optimize the extraction of renewable energy, MPPT algorithms must be very accurate. Numerous systems, including FSSO, CSO, SSA, WOA, and GWO, closely monitor the maximum power point. Power oscillation is a measure of stability. All of the technologies produce a steady stream of energy, with only a few fluctuations in production. The complexity of computer resources and algorithms is particularly sensitive. FSSO, CSO, SSA, WOA, GWO, and DE fall into the moderate to high complex category due to their sophisticated algorithms and processing requirements. The FSSO, CSO, SSA, and WOA are robust and capable of withstanding unusual working conditions. Because of their moderate to poor resilience, PSO and DE are susceptible to changes in the environment or inside the system. When supporting a wide range of system sizes and configurations, scalability, adaptability, and versatility are crucial. The scalability, adaptability, and diversity of FSSO, CSO, SSA, WOA, and GWO make them appropriate for numerous PEMFC systems and applications. When compared, PSO and DE offer more flexibility, variety, and modest scalability. In conclusion, the entire analysis highlights the benefits and drawbacks of each MPPT technique, enabling informed choices based on project requirements and limitations.

**Table 7:** The PEMFC parameters.

PEMFC parameter	Definition	Values
$\xi_{1}$	All cell model variable values	$- 0.514$
$\xi_{2}$	All cell model variable values	${0}{\text{.00286 + (0}}{\text{.0002*ln( A)) + (4}}{\text{.3*ln( C}}_{{{\text{H2}}}} {)*1 } \times { 10}^{ - 5} {)}$
$\xi_{3}$	All cell model variable values	$0.41 \, \times {10}^{ - 4}$
$\xi_{4}$	All cell model variable values	$- 0.93 \, \times \, 10^{ - 4}$
${\text{C}}_{{{\text{H2}}}}$	Hydrogen concentration	${\text{P}}_{{{\text{H2}}}} {/(1}{\text{.1}} \times {10}^{ - 6} {\text{)*exp( - 77/T)}}$
${\text{C}}_{O2}$	Oxygen concentration	$P_{O2} /(5.08 \times 10^{ - 5} *\exp (( - 498)/T))$
$P_{H2}$	Hydrogen pressure	$1.5\;{\text{atm}}$
$P_{O2}$	Level of oxygen pressure	$1\;{\text{atm}}$
$A$	A field of fuel cell activity	70 cm^2^
$J$	Concentration of current	$\frac{{I_{FC} }}{A}$
$T$	Maximum allowable temperature for fuel cell operation	$328\;({\text{Kalven}})$
$J_{\max }$	The most excellent density of electric current	1.5 A/cm^2^
$B$	A variable that is dependent on the kind of energy source (PEMFC) and how it is used	0.0016 V
$\lambda$	Molecules' amount of water	$27.7$

**Table 8:** Power converter (Boost) parameter.

Component	Capacitance (C)	Resistance (R)	Inductance (L)	Switching frequency
Value	${1 } \times \, 10^{ - 6} {\text{ F}}$	${\text{26 Ohms}}$	${98 } \times \, 10^{ - 2} {\text{ H}}$	${\text{2 KHZ}}$

## Conclusion and future research directions

Comparing the Flying Squirrel Search Optimization (FSSO) and Cuckoo Search Optimization (CSO) algorithms for Maximum Power Point Tracking in PEM fuel cells gives us useful information about how well they work in different situations, as shown in Table 4. Typically, FSSO exhibits a quick response time when considering various optimization variables. For example, the power output of FSSO demonstrates a response time of 0.5 s, which is slightly quicker than CSO's 0.6 s. In the same manner, FSSO demonstrates a reaction time of 0.5 s for both current and voltage outputs, which are CSO's response times. Nevertheless, when exposed to changes in pressure and temperature, FSSO consistently exhibits quicker response times in comparison to CSO. When pressure changes, FSSO's reaction time decreases to 0.3 s, but slightly increases to 0.32 s when temperature changes. On the other hand, the CSO reaction time varies more noticeably, decreasing to 0.35 s with changes in temperature but increasing significantly to 0.84 s when both pressure and temperature change simultaneously. Furthermore, it is crucial to emphasize the significant maximum power output (P_max_) that both algorithms attain under various conditions. Under typical circumstances, the FSSO typically achieves a maximum power output of 1990.68686017 W, but it steadily increases to 2064.526663 W when pressure and temperature change. Similar to the FSSO, the CSO also shows a steady increase in Pmax, starting at 1990.68686014 W under normal conditions and reaching 2064.5266605 W with adjustments to pressure and temperature. The comparison between FSSO and CSO highlights their unique advantages and compromises. The FSSO demonstrates enhanced sensitivity to variations in temperature and pressure because of its accelerated response time, enabling it to achieve slightly higher levels of power generation at its peak. When deciding between FSSO and CSO, it is important to consider the specific needs and priorities of the PEM fuel cell system, taking into account the trade-off between efficiency and reactivity. Overall, both FSSO and CSO methods show great potential for enhancing power generation in PEM fuel cell devices across various outside circumstances.

Future research directions for optimizing PEM fuel cell systems through MPPT techniques include exploring hybrid optimization approaches combining FSSO and CS algorithms, conducting experimental validations in real-world fuel cell structures, developing advanced dynamic models to capture system complexities, enhancing fault tolerance and robustness against disturbances, integrating MPPT with energy storage systems for improved energy management, extending applications beyond automotive sectors, investigating machine learning approaches for adaptive control, and conducting comprehensive environmental impact assessments. These endeavors aim to push the boundaries of fuel cell optimization, ensuring greater efficiency, reliability, and sustainability in various applications and industries.

## Data Availability

The datasets used and/or analysed during the current study available from the corresponding author on reasonable request.
